# Retraction: The impact of newly synthesized sulfonamides on soil microbial population and respiration in rhizospheric soil of wheat (*Triticum aestivum* L.)

**DOI:** 10.1371/journal.pone.0272421

**Published:** 2022-08-17

**Authors:** The PLOS ONE Editors

The *PLOS ONE* Editors retract this article [1] because it was identified as one of a series of submissions for which we have concerns about authorship, competing interests, and peer review. We regret that the issues were not addressed prior to the article’s publication.

AS, AZ, MZA, SH, MTK, MZ, ATKZ, and KMA did not agree with the retraction. SMA either did not respond directly or could not be reached.
